# Effects of Tai Chi on Biomarkers and Their Implication to Neurorehabilitation – A Systemic Review

**DOI:** 10.1093/geroni/igab046.3252

**Published:** 2021-12-17

**Authors:** Howe Liu, Yasser Salem

**Affiliations:** 1 University of North Texas Health Science Center, Fort Worth, Texas, United States; 2 University of North Texas Health Science Center/, Fort Worth, Texas, United States

## Abstract

Introduction: As an effective holistic therapeutic exercise program, Tai Chi (TC) has been widely used for patients with a variety of neurological disorders. In last 1-2 decades, there has been an increase in the number of research studies that examined the TC effects on biomarkers andIntroduction: As an effective holistic therapeutic exercise program, Tai Chi (TC) has been widely used for patients with a variety of neurological disorders. In last 1-2 decades, there has been an increase in the number of research studies that examined the TC effects on biomarkers including inflammatory cytokines, oxidative stressors, and neurotrophic factors. Thus, the purpose of this article is to review such effects and their possible implications to neurorehabilitation. Method: In this systematic review, we searched TC-related articles from the last 15 years until July 2020 that had investigated changes of biomarkers after TC practice. The search identified 24 studies that were included in our analysis. Results: It is found that TC practice is able to 1) reduce pro-inflammatory and increase anti-inflammatory cytokines (including Interleukins -1, 6, 10, 12, tumor necrosis factor, the nuclear factor kappa-light-chain-enhancer of activated B cells, and the C-reactive protein); 2) decrease oxidative stress factors (like plasma 8-isoprostane, malondialdehyde, and protein carbonylation); and 3) increase neurotrophic factors (brain-derived neurotrophic factor (BDNF), and N-Acetylaspartate). Conclusions: TC may take effect on patients with neurological dysfunctions through anti-inflammation, anti-oxidative stress, and neural health promotion.

